# Nutritional and Technological Quality of High Protein Pasta

**DOI:** 10.3390/foods10030589

**Published:** 2021-03-11

**Authors:** Maria Cristina Messia, Francesca Cuomo, Luisa Falasca, Maria Carmela Trivisonno, Elisa De Arcangelis, Emanuele Marconi

**Affiliations:** Department of Agricultural, Environmental and Food Sciences (DiAAA), University of Molise, Via F. De Sanctis snc, 86100 Campobasso, Italy; messia@unimol.it (M.C.M.); francesca.cuomo@unimol.it (F.C.); falasca@unimol.it (L.F.); mariacarmela.trivisonno@unimol.it (M.C.T.); elisa.dearcangelis@unimol.it (E.D.A.)

**Keywords:** pasta, unconventional ingredients, nutritional value, cooking quality, legumes, *Spirulina Platensis*

## Abstract

Pasta has an important role in human nutrition for its high content of complex carbohydrates and its widespread use. It can be an efficient delivery system or carrier of non-traditional raw material, including additional health-promoting ingredients. The partial replacement of semolina with high-protein raw materials leads to the improvement of the biological value of pasta proteins. In order to obtain pasta with high nutritional protein value and with excellent cooking properties, various recipes have been formulated with different percentages of semolina and unconventional high-protein raw materials (peas and soy isolate proteins, egg white, whey proteins and *Spirulina platensis*). High-protein pasta was produced using a pasta making pilot plant and the nutritional quality (protein content and quality) and sensorial properties were assessed. All experimental pastas showed optimal performances. Pasta prepared with pea protein isolate, whey proteins and *Spirulina platensis* showed improved chemical score and digestible indispensable amino acid scores, an eye-catching color, and an excellent cooking quality.

## 1. Introduction

Pasta is a foodstuff with an important role in human nutrition. It is popular with consumers for its easy handling, storage and preparation. In addition to its high content of complex carbohydrates with low glycaemic index, pasta also contains proteins. The quality of a protein is substantially related to its composition in essential amino acids and its digestibility. High-quality proteins contain all the essential amino acids at levels equal to or higher than those of the reference amino acid pattern of FAO/WHO/UN [1]. On the contrary, nutritionally incomplete or low biological value proteins are those lacking or deficient in one or more essential amino acids. All proteins of animal origin, with the exception of collagen, are considered complete proteins, while vegetable proteins, with a few exceptions, have a relative deficiency in certain essential amino acids. The limiting amino acid is the essential amino acid present in a protein in the lowest quantity, thus arginine is the limiting amino acid for casein, methionine for fish and egg proteins, and lysine is the amino acid of which commonly vegetable proteins are more deficient, especially those of cereals and cereal-based foods, such as pasta. In this context, the chemical score (CS) is the parameter used to describe the quality of proteins in terms of the potential ability of the dietary protein to provide the appropriate amount of essential amino acids. Proteins with a CS close or equal to 100 are considered better nutritionally, and therefore able to adequately meet human needs.

The interest in protein supplements is particularly exhibited by athletes and by people who need to bring a greater amount of protein into their diet. The protein requirement of the adult can increase significantly with sporting activity, starting from a base value, indicated in the LARN (Livelli di Assunzione di Riferimento di Nutrienti ed energia) [2], of 0.8 g/kg (protein/kg of body weight), useful for the sedentary subject, to values higher than 1.5 g/kg, indicated to cover the athlete’s needs for the growth, maintenance, and repair of muscle [3,4,5,6,7,8]. However, it should be noted that adopting a diet very rich in proteins can lead to a reduction in carbohydrate intake, since sportspeople usually adhere to strict and scrupulous daily energy intakes. This imbalance in the diet could be counterproductive and potentially harmful in terms of health. There is a widespread belief among body builders that a high-protein diet, further integrated with purified proteins, is the fundamental factor for the development of muscle mass. The protein requirement increases if the training is aimed at developing strength and therefore muscle trophism, or if the training load is particularly intense. The use of nutritional supplements is very widespread among athletes of different levels, although the scientific literature does not report any data on their functions and their effects, which are instead promoted to the public. However, regardless of sportsmen, consumers are currently changing their eating habits thanks to the greater awareness they are acquiring regarding the well-being that a diet rich in foods of plant origin and low in foods of animal origin brings, both to the body and to the environment [9]. As shown by Ranganathan and coworkers [10], it is more expensive to obtain animal resources than plant-based ones. This aspect represents one of the main reasons for orienting new food styles towards other sustainable and effective sources able to provide high-quality food production while coping with population growth.

The commercial segment of high-protein pasta, driven by particular nutritional needs but also by food trends, has been growing in recent years. The nutritional needs required by consumers (higher protein content) have been met by the food industry, as demonstrated by the numerous products on the market. Innovative pasta recipes, including the replacement of semolina with alternative ingredients, have already been proposed, and non-traditional raw materials, soybean, pea, bean, chickpea flours or isolates, but also milk products such as whey proteins, casein and powdered milk, could be used [11,12,13]. In particular, legumes represent an interesting source of nutrients (protein, minerals, fiber) [14], and represent a valid ingredient in the development of diets that are healthy to humans and sustainable for the environment, since they can help to mitigate environmental climate change by reducing the carbon and water footprint [15]. Therefore, there has been an increasing interest in integrating legumes into food production. Although legumes’ proteins are relatively low in sulfur amino acids and tryptophan, they have high lysine contents. Consequently, legumes and cereals are nutritionally complementary. The partial replacement of semolina with legume flours in the preparation of pasta leads to the improvement of the protein biological value and the amino acid CS.

More recently, microalgae and cyanobacteria such as *Chlorella* spp., *Dunaliella* spp., and *Spirulina* spp. are becoming more popular as new, highly nutritious food ingredients [16,17,18]. *Spirulina platensis* (spirulina) is rich in digestible protein, fat with unsaturated fatty acids, mineral, clorophyll and B group vitamins, in particular B12 vitamin [19,20]. For its characteristics, spirulina has been proposed for different food preparations, such as yogurt [21], snacks [22], or in Indian recipes [23] for the development of functional foods. Moreover, some studies have demonstrated the potential of this microalgae in the prevention and treatment of diseases related to metabolic syndrome [24]. Recently, a study has been published wherein spirulina has been used as a filling, together with other ingredients, in stuffed pasta [25]. The investigation was mainly focused on the acceptance of consumers of spirulina, and it emerged that its taste is accepted only in small amounts. Another recent study [26] used spirulina encapsulated in alginate microcapsules in the production of fresh pasta prepared with wheat flour. Encapsulation partially protects spirulina from the loss of its antioxidant potential, and the pasta presented green dots of a non-uniform color on the surface, which did not negatively influence the consumer’s judgement.

Using legumes and spirulina as food ingredients represents an opportunity to reconcile the food system with the needs of the planet, and to encourage a healthy and balanced diet with beneficial effects for both humans and the environment, as indicated in the latest European strategies on the agri-food system “From Farm to Fork Strategy—For a fair, healthy and environmentally friendly food system and Green Deal” [27].

However, the amount of high-protein material that can be added to or substituted for semolina represents a compromise between nutritional improvement and the achievement of satisfactory sensory and functional properties in pasta. Often, improving protein quantity and quality in pasta by the addition of various raw materials from plant or animal sources can lead to a decrease in pasta’s sensory and cooking qualities.

Based on the above considerations, here, soy protein isolate, pea protein isolate, whey proteins, and spirulina were proposed as additional ingredients to improve the nutritional quality of semolina pasta. The ingredients were used to produce high-protein pasta, and the effects on the nutritional and cooking quality properties were investigated and compared with 100% semolina pasta.

## 2. Materials and Methods 

### 2.1. Ingredients

Soy protein isolate (ABS FOOD srl, Peraga di Vigonza (PD), Italy), peas protein isolate (ABS FOOD srl, Peraga di Vigonza (PD), Italy), egg white (EUROVO srl, Bologna, Italy), whey proteins (Volac International Ltd., Hertfordshire, UK), high-quality durum wheat semolina (high protein, gluten index = 95) from a local distributor, and *Spirulina platensis* (spirulina) (ATI Biotech, Napoli, Italy) were used as ingredients to develop high-protein pasta.

### 2.2. Commercial Pasta

Five short commercial pastas (“rigatoni” and “penne” shape) with high protein contents were purchased in a local supermarket. The protein content and the ingredients listed on their label are shown in Table 1.

### 2.3. Proximate Composition 

Moisture, ash and lipid content were determined according to the ICC methods 109/1, 104/1 and 136, respectively [28]. The dietary fiber was determined according to the AACC method 32.05 [29]. Protein content (N × 6.25) was determined according to the Dumas combustion method (AACC method 46–30) [29], using a Leco nitrogen determiner, model FP 528 (Leco Corp., St. Joseph, MI, USA).

### 2.4. Amino Acids Analysis and Chemical Score

Amino acids were analyzed after acidic and alkaline hydrolysis. Acidic hydrolysis: a sample, corresponding to 25 mg of protein, was hydrolyzed with 25 mL of 6 N HCl at 110 °C for 24 h. Afterwards, the sample was cooled, filtered, evaporated to dryness and re-dissolved in 0.1 N HCl. Alkaline hydrolysis for tryptophan: a sample containing 10 mg of protein was added to 1 mL of distilled water, shaken, supplemented with 10 N NaOH (5 mL) and distilled water (4 mL), and then hydrolyzed for 18 h at 110 °C. After cooling, the sample was neutralized by adding 6 N HCl, evaporated to dryness, and re-dissolved in 0.1 N HCl. Before analysis, all samples were diluted 1:50–1:100 with ultra-pure water, and analyzed by an ICS6000 chromatographic system (Thermo Fisher Scientific S.p.A, Milano, Italy). Separation was performed with an Aminopac PA10 analytical column (250 × 2 mm, 8.5 μm particle size) (Thermo Fisher Scientific S.p.A, Milano, Italy). Chromatographic separation of the amino acids was performed according to the following conditions (Table 2).

Chemical score (CS) and digestible indispensable amino acid score (DIAAS%) were calculated according to the Food and Agriculture Organization [1] using the recommended amino acid scoring pattern for older children, adolescents and adults. 

### 2.5. Pasta Making

Short pasta (rigatoncini shape) was manufactured through an experimental pasta making apparatus (NAMAD, Roma, Italy) composed of a press and a dryer following the approved method 66–41 [29]. The press (capacity 10–20 kg) was equipped with a vacuum-mixing and -extruding system, as well as with a water-cooling jacket for the barrel and the extrusion head to reduce heat and to maintain a constant extrusion temperature lower than 50 °C. The static dryer was equipped with a heat ventilator unit, to ensure uniform temperature and ventilation in all parts of the apparatus, and a moisture control unit. Semolina and other ingredients were mixed for 15 min with tap water (30 °C) to obtain a dough suitable for extrusion. Extrusion occurred at 30 ± 2 °C and at a pressure of 76 ± 5 bar. Each series of short pasta was dried at a maximum temperature of 50 °C for 24 h. At the end of the drying cycle pasta was conditioned at room temperature (~20 °C) for 24 h.

### 2.6. Pasta Characterization

Optimum cooking time, firmness (by chewing), liveliness (by manual handling) and starch release (by manual handling) were determined according to International Standard ISO 7304-1 [30]. A rating scale ranging from 10 to 100 was used (Table 3). A panel of 10 trained judges was used to assess pasta characteristics. The total score was calculated by adding together the ratings obtained for firmness, liveliness and starch release and then dividing the sum by 3.

### 2.7. Statistical Analysis

Data reported for all parameters are the average values of measurements obtained from the analysis of three different aliquots of each sample, and were expressed as mean ± standard deviation (mean value ± sd). Analysis of variance (ANOVA) and Tukey HSD tests were performed on the protein content and cooking quality scores of the experimental pastas using RStudio version 1.2.5033 (RStudio Team (2019). RStudio: Integrated Development for R. RStudio, Inc., Boston, MA, USA, http://www.rstudio.com/ (accessed on 3 March 2021)). Significant differences were set for *p* < 0.05. 

## 3. Results

### 3.1. Cooking Quality Assessment of Commercial High-Protein Pasta

The nutritional needs of consumers are met by the food industry, as demonstrated by numerous products on the market. Five commercial pastas with a high protein contents (ranging between 40 and 65%) were checked for composition and ingredients. As shown in the list of ingredients (Table 1), the abovementioned pastas included a wide range of ingredients such as soy protein, pea protein, lentils flour, eggs, egg white, inulin, caseinate and gluten, in addition to the presence of additives such as E412 (guar gum) and E401 (sodium alginate), and it emerged that they were prepared by reducing or annihilating the percentage of durum wheat semolina used in the formulation. Moreover, food additives, such as guar gum are commonly used as structuring agents with the aim of replacing the gluten that is missing in the alternative ingredients used to increase the protein content. Although commercial pastas satisfy the requirement related to the high protein content, they are not always able to satisfy the consumer from a sensorial point of view. 

### 3.2. Characterization of Raw Materials for High-Protein Pasta Production

Durum wheat semolina, due to the rheological properties of its proteins (gluten) and the high content of pigments, is considered the best raw material for pasta making. The preparation of pasta with unconventional ingredients is challenging due to the absence/reduced formation of the protein network that prevents the disintegration of the pasta during cooking. In this experimentation, the possibility of using semolina in combination with other high protein raw materials to produce pasta with high protein content, improved protein quality and optimum cooking quality was studied. In order to only partially replace the semolina, among the possible ingredients, we tested protein isolates of soy and pea, egg white, whey proteins and spirulina.

To obtain excellent quality pasta, without the adjuvants/additives (e.g., guar gums, sodium alginate) generally enclosed in the recipes of commercial pasta, high-protein and high-gluten semolina was used. To counteract the lack of gluten and help the formation of a cohesive mass, we chose to add egg white and whey proteins, as widely reported in the literature [31,32].

The proximate composition of semolina and unconventional raw materials used for the production of experimental high-protein pasta is reported in Table 4. 

For the selected raw material, the amino acids content, the CS, and the definition of the limiting amino acid, calculated on the basis of the Food and Agriculture Organization’s described pattern [1], were assessed (Table 5).

### 3.3. Definition of Pasta Recipes and Pasta Cooking Quality 

Different formulations for high-protein pasta were hypothesized in order to produce pasta with a protein content above 40% and an improved CS (90–100%) compared to 100% semolina pasta. The formulations were studied, taking into account the costs of raw materials, and including ingredients at appropriate quantities to obtain a final product with acceptable sensorial characteristics and good cooking quality. Moreover, salt was not added to ensure that the salt in the pasta was due only to the sodium naturally contained in the ingredients, and also to meet the needs of consumers as regards preventing arterial hypertension. Spirulina (1%) as a source of proteins and color was also included among the ingredients for pasta production (formulation FHP2, FHP4, FHP6 and FHP8).

The hypothesized formulations are shown in Table 6.

High-protein short pastas (“rigatoncini” shape) were produced, starting from the formulations in Table 6. The 100% semolina pasta was used as the control sample. The results for the protein content, chemical score, DIAAS%, optimal cooking time and cooking quality of all produced pastas are reported in Table 7.

## 4. Discussion

The nutritional quality of a protein that is deficient in essential amino acids can be improved by suitable supplementation with other proteins rich in essential amino acids. Therefore, the addition of proteins from other sources in cereal-based formulations results in a complete and balanced level of essential amino acids.

The heterogeneity of raw materials (protein can come from both plant and animal sources) potentially usable in the production of cereal-based foods, and the replacement of all or part of the conventional flours with other cereals or ingredients different from cereals, often entails the need to make changes to the traditional production process. Balanced formulations and adequate technological processes must be adopted to compensate for any changes in functional properties caused by the incorporation of new ingredients [12,32,33].

The introduction of unconventional material in the recipe of commercial pasta (Table 1) definitely increased the protein content, especially if these are added at high levels. However, this impacted the quality attributes of pasta. In fact, besides a high protein content, the panelists’ observations about smell, taste, texture and color were quite negative. To overcome this problem, with the aim of achieving optimal technological behavior that could have a positive influence on the cooking quality of the pasta, the formulations for innovative pasta were hypothesized, maintaining a higher percentage of semolina than the remaining ingredients (Table 6). The loss of firmness following the cooking caused by gluten deficiency, and the possible presence of unpleasant tastes and flavors due to alternative ingredients such as legumes, would prejudice the consumer’s acceptance of the pasta. In fact, pasta compounds such as proteins, fat, and carbohydrates can absorb legumes’ flavor compounds, resulting in their retention [34]. It thus means that the amount of pulse ingredient to add will be limited by flavor characteristics. Up to a certain percentage, the “off” flavor of the pulse ingredient can be masked by other compounds present in the food matrix [35]. 

To assess the improvement of the nutritional characteristics and the effects of the unconventional raw material’s addition on the cooking quality of pasta, the protein content, the CS and the cooking quality were evaluated. Data in Table 7 show that all the experimental pasta had good protein contents (40.7–54.7% fresh weight, f.w.), an improved CS (CS = 91–100) compared to the pasta with 100% semolina (CS = 44), and excellent cooking quality (total score between 83.3 and 96.7). To assess the protein quality of pasta, besides CS, the DIAAS% was calculated. The actual capacity of protein to satisfy the amino acid needs requires the use of corrections for amino acid digestibility and availability. The FAO [1] recommendation is to use DIAAS as the measure of protein quality, rather than measures such as the protein efficiency ratio (PER). A nutritional claim for protein content (i.e., “source”, “high” according to Regulation (EC) No 1924/2006 [36]) should be coupled to the computing of the DIAAS values, to discriminate the quality of the protein itself. For excellent/high-protein quality, DIAAS ≥100 were proposed, for good/source values ranging from 75 to 99, while it was stated that no claim should be allowed for the cut-off value of, e.g., 75. All the recipes proposed in this experimentation led to the production of pasta with DIAAS higher than 100, showing the good combinations used to achieve high-quality protein in the final product.

The presence of a high content of semolina, egg white and whey proteins ensured the structuring of the pasta and therefore the cooking quality, achieving an evaluation comparable to the 100% semolina pasta used as a control. Pasta produced using formulation FPH7 and 8 in addition to having a protein content of 41.1% f.w. and optimal protein quality (CS = 100, DIAAS = 100) higher than the control, among all the experimental pasta, showed the highest score for cooking quality. The results have shown that it is possible to produce nutritionally valid pasta with excellent cooking quality by only partially replacing the semolina and without adding adjuvants/additives, as used in and shown on most commercial pasta labels. Moreover, according to Regulation (EC) No 1924/2006 [36] and Commission Regulation (EU) No 432/2012 [37], all the pastas of this experimentation can boast both the nutrition claims (source of protein, high protein) and the health claims related to proteins, because they are foods that are at least a source of protein, as referred to in the claim source of protein, as listed in the Annex to Regulation (EC) No 1924/2006 [36].

**Figure 1:**
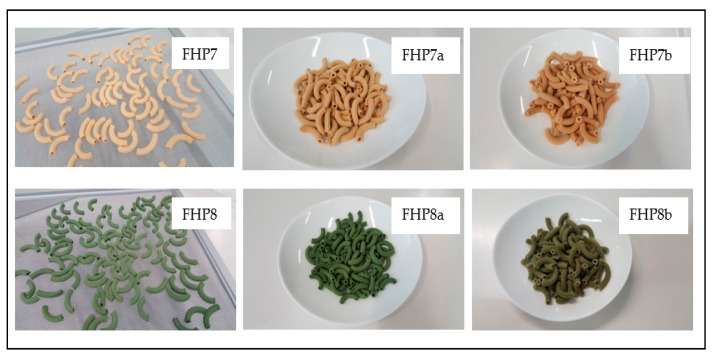
Raw (a) and cooked pasta (b) produced using formulation 7 (FHP7) and 8 (FHP8).

Moreover, the presence of spirulina not only contributed to increasing the protein content, but in combination with pea protein and whey proteins, also determined a higher pasta firmness. According to Fradique et al. [38], the reinforcement of the gluten network could cause an extra establishment of disulfide bonds, formed between the sulfhydryl groups of cysteine residues in gluten proteins. Moreover, all pasta produced with spirulina showed an intense green color (Figure 1), which is less usual for pasta consumers, but this did not affect its acceptability because its taste and flavor were ordinary. The intense green color maintained by the pasta after the cooking (Figure 1) is also due to the fact that spirulina presents only chlorophyll *a* in its constitution, which is more stable under thermal processes than the chlorophyll *b* of vegetables, including the spinach generally used to color the products [39]. Similar observations have also recently been reported by Mostolizadeh et al. [40] using spirulina in pasta production. 

## 5. Conclusions

The study highlights the suitability of unconventional raw materials, such as legumes, whey proteins and spirulina, for obtaining pastas improved in terms of protein content, amino acids chemical score and cooking quality. 

Good quality semolina (high protein, gluten strength) and its limited substitution with other ingredients is the key to obtaining pasta with a high cooking quality. Through suitable formulations it is possible to obtain not only the protein-enriched pasta, but also the amino acid complementarity useful for carrying out physiological functions and for ensuring the production of pasta with high-quality proteins. Spirulina, although in small concentrations, contributed to improving protein content, positively affecting the pasta firmness and leading to a green color in the product, which was stable even after processing and cooking. 

Examples of fortified pasta produced in this experimentation may help to broaden the offer for people who want to improve the nutritional quality of their diet or satisfy the particular needs of sportsmen.

## Figures and Tables

**Table 1:** Protein content and ingredients of commercial high-protein pasta (CP).

Sample	Protein (%)	Ingredients
CP1	60	soy protein isolate, wheat flour, gluten, egg white, pea protein, wheat fiber, inulin, guar gum
CP2	40	semolina, pea protein, egg
CP3	60	gluten, wheat flour, soy protein isolate, egg white powder, whey proteins, pea protein, wheat fiber, guar gum
CP4	52	vegetal proteins (soy, pea), semolina, egg white, sodium alginate, L-methionine, L-threonine
CP5	65.1	soy protein, lentil flour, pea protein, calcium caseinate, egg

**Table 2:** Conditions for chromatographic separation of amino acids.

Mobile Phase (0.250 mL/min)				Time/Potential Waveform		
Time (min)	H_2_O (%)	NaOH (%)	NaOAc (%)	Time (sec)	Potential (V)	Integration
0.0	80	20	0	0.00	+0.13	
2.0	80	20	0	0.04	+0.13	
12.0	80	20	0	0.05	+0.28	
16.0	68	32	0	0.11	+0.28	began
24.0	36	24	40	0.12	+0.60	
40.0	36	24	40	0.41	+0.60	
40.1	20	80	0	0.42	+0.28	
42.1	20	80	0	0.56	+0.28	end
42.2	80	20	0	0.57	−1.67	
62.0	80	20	0	0.58	−1.67	
				0.59	+0.93	
				0.60	+0.13	

**Table 3:** Rating scale for pasta sensory analysis [30].

Firmness	Liveliness	Starch Release
100—very high (very firm)	100—very high (not at all sticky)	100—very low (no starch)
80—high	80—high	80—low
50—medium	50—medium	50—medium
30—low	30—low	30—high
10—very low (very tender)	10—very low (very sticky)	10—very high (large quantity of starch)

**Table 4:** Proximate composition (g/100 g d.w.) of wheat semolina and other raw materials.

Sample	Protein	Lipid	Ash	Carbohydrates *	Fiber
Wheat Semolina	14.3 ± 0.01	1.5 ± 0.02	0.8 ± 0.00	79.2	4.2 ± 0.30
Soy Protein Isolate	90.0 ± 0.21	1.1 ± 0.01	6.5 ± 0.11	1.3	1.1 ± 0.23
Pea Protein Isolate	87.9 ± 0.05	2.0 ± 0.02	6.9 ± 0.21	1.4	1.8 ± 0.43
Egg White	89.2 ± 0.12	0.0 ± 0.00	8.3 ± 0.05	0.0	0.0 ± 0.00
Whey Proteins	91.8 ± 0.81	0.4 ± 0.00	2.1 ± 0.23	5.7	0.0 ± 0.00
Spirulina	58.5 ± 0.32	8.9 ± 0.15	7.3 ± 0.45	20.8	4.5 ± 0.30

* Calculated by difference.

**Table 5:** Amino acid (g/100 g protein), chemical score (CS) and limiting amino-acid of soy protein, peas protein, egg white, whey proteins and wheat semolina.

Essential Amino Acids	Soy Protein Isolate	Peas Protein Isolate	Egg White	Whey Proteins	Wheat Semolina	FAO [1] Amino Acid Scoring Patterns (mg/g)
Histidine	2.29 ± 0.13	3.50 ± 0.09	2.20 ± 0.05	2.13 ± 0.03	2.05 ± 0.11	16
Isoleucine	3.92 ± 0.22	1.65 ± 0.08	5.35 ± 0.09	7.12 ± 0.10	3.69 ± 0.07	30
Leucine	7.49 ± 0.01	7.80 ± 0.08	8.06 ± 0.30	11.35 ± 0.55	7.07 ± 0.05	61
Lysine	5.69 ± 0.14	6.63 ± 0.31	6.78 ± 0.21	9.83 ± 0.13	2.09 ± 0.24	48
Methionine	1.20 ± 0.04	0.25 ± 0.22	3.94 ± 0.10	2.25 ± 0.09	1.56 ± 0.08	Methionine + Cysteine 23
Cysteine	1.84 ± 0.04	1.48 ± 0.09	2.92 ± 0.05	2.51 ± 0.21	2.45 ± 0.07	
Phenylalanine	4.86 ± 0.15	5.99 ± 0.12	5.85 ± 0.15	3.44 ± 0.28	4.89 ± 0.19	Phenylalanine + Tyrosine 41
Tyrosine	3.41 ± 0.22	2.68 ± 0.08	4.19 ± 0.19	3.42 ± 0.56	2.56 ± 0.09	
Threonine	3.77 ± 0.00	3.19 ± 0.10	4.67 ± 0.09	7.72 ± 0.02	2.77 ± 0.04	25
Valine	3.98 ± 0.07	4.64 ± 0.13	6.84 ± 0.10	6.65 ± 0.23	4.33 ± 0.01	40
Tryptophan	0.51 ± 0.02	1.15 ± 0.09	1.60 ± 0.07	1.93 ± 0.04	0.98 ± 0.02	6.6
Chemical Score (CS)	77	79	100	100	44	
Limiting Amino acid	Tryptophan	Sulphur amino acid (Met + Cys)	--	--	Lysine	

**Table 6:** Hypothesized high-protein pasta formulations (FHP).

Formulation	Ingredients (%)					
	Wheat Semolina	Soy Protein Isolate	Pea Protein Isolate	Egg White	Whey Proteins	Spirulina
FHP0	100	0	0	0	0	0
FHP1	44	17.4	30.8	7.8	0	0
FHP2	43	17.4	30.8	7.8	0	1
FHP3	38	25	25	12	0	0
FHP4	37	25	25	12	0	1
FHP5	45	23	20	12	0	0
FHP6	44	23	20	12	0	1
FHP7	60	0	30	0	10	0
FHP8	59	0	30	0	10	1

**Table 7:** Cooking quality of experimental high-protein pastas (Pasta HP).

Pasta	Protein (%)	Chemical Score	DIAAS (%)	Optimal Cooking Time (min)	Cooking Quality				
					Firmness	Liveliness	Starch Release	Total Score	Panel Comments
Pasta HP0	12.4 ± 0.01 ^h^	44	36	13′:00″	100 ± 0.0 ^a^	90 ± 4.71 ^a^	90 ± 4.08 ^b^	93.3	Amber/yellow color, pleasant smell and taste, optimal consistency
Pasta HP1	50.6 ± 0.02 ^c^	91	100	14′:00″	100 ± 0.0 ^a^	80 ± 5.27 ^b^	80 ± 3.33 ^c^	86.7	Light brown color, pea flavor, excellent consistency
Pasta HP2	50.9 ± 0.04 ^b^	91	100	14′:50″	100 ± 0.0 ^a^	90 ± 4.08 ^a^	80 ± 3.33 ^c^	90.0	Green color, pea smell, excellent consistency
Pasta HP3	54.5 ± 0.10 ^a^	100	100	15′:00″	80 ± 2.36 ^c^	80 ± 5.27 ^bc^	90 ± 6.24 ^b^	83.3	Light brown color, mild pea flavor, good consistency
Pasta HP4	54.7 ± 0.05 ^a^	100	100	15′:00″	90 ± 7.07 ^b^	85 ± 4.71 ^ac^	90 ± 4.08 ^b^	88.3	Green color, slight herbaceous flavor, good consistency
Pasta HP5	50.1 ± 0.11 ^e^	95	100	14′:30″	100 ± 0.0 ^a^	85 ± 4.71 ^ac^	80 ± 4.71 ^c^	88.3	Light brown color, pleasant taste, optimal consistency
Pasta HP6	50.4 ± 0.05 ^d^	95	100	15′:00″	100 ± 0.0 ^a^	87 ± 5.87 ^a^	80 ± 2.36 ^c^	89.0	Green color, pleasant flavor, optimal consistency
Pasta HP7	40.3 ± 0.00 ^g^	100	100	15′:30″	97.5 ± 4.24 ^a^	90 ± 4.08 ^a^	100 ± 0.00 ^a^	95.8	Light brown color, optimal consistency
Pasta HP8	40.7 ± 0.01 ^f^	100	100	15′:30″	98 ± 2.58 ^a^	92 ± 5.87 ^a^	100 ± 0.00 ^a^	96.7	Green color, optimal consistency

Different letters in a column indicate statistically significant differences (*p* < 0.05).

## Data Availability

The data presented in this study are available on request from the corresponding author.
